# Associations between migraine and major cardiovascular events in type 2 diabetes mellitus

**DOI:** 10.1186/s12933-022-01705-3

**Published:** 2022-12-09

**Authors:** Dae Young Cheon, Kyungdo Han, Ye Seul Yang, Yerim Kim, Sang-Hwa Lee, Chulho Kim, Jong-Hee Sohn, Mi Sun Oh, Byung-Chul Lee, Minwoo Lee, Kyung-Ho Yu

**Affiliations:** 1 Division of Cardiology, Department of Internal Medicine, Dongtan Sacred Heart Hospital, Hwaseong, Korea; 2 Department of Statistics and Actuarial Science, Soongsil University, Seoul, Korea; 3 Department of Medicine, Seoul National University College of Medicine, Seoul, Republic of Korea; 4 Department of Neurology, Kangdong Sacred Heart Hospital, Seoul, Korea; 5 Department of Neurology, Chuncheon Sacred Heart Hospital, Chuncheon, Korea; 6 Department of Neurology, Hallym University Sacred Heart Hospital, Anyang, Korea

**Keywords:** Migraine, Diabetes mellitus, Stroke, Myocardial infarction, Cardiovascular death

## Abstract

**Background:**

Migraine is one of the most common primary headache disorders and a well-known risk factor for cardiovascular disorders. We aimed to investigate the association between migraine and major cardiovascular outcomes, including myocardial infarction (MI), ischemic stroke (IS), and cardiovascular death (CVD) in people with type 2 diabetes.

**Research design and methods:**

A total of 2,229,598 people from the nationwide Korean National Health Insurance Service database with type 2 diabetes but without a previous history of MI and IS were included in this study. We identified patients over 20 years of age with migraine using the claim data of International Statistical Classification of Diseases Related Health Problems, Tenth Revision (ICD-10) code G43. The patients with migraine were divided according to their migraine aura status.

**Results:**

Migraine was present in 6.3% of the study population. Cases observed for MI, IS, CVD, and all-cause death were 2.6%, 3.6%, 5.9%, and 7.9%, respectively. The diagnosis of migraine was significantly associated with an increased risk of MI, IS, and CVD. The results remained significant after adjusting for covariates, including age, sex, body mass index, alcohol intake, smoking habits, physical activity, economic status, hypertension history, dyslipidemia, and duration of type 2 diabetes (MI, adjusted hazard ratio [aHR]: 1.182, 95% confidence interval [CI]: 1.146–1.219; IS, aHR: 1.111, 95% CI 1.082–1.14; CVD, aHR: 1.143, 95% CI 1.12–1.167). In particular, the presence of aura was associated with a higher risk of MI development compared to the non-aura group. The difference became more prominent with progressing age.

**Conclusions:**

In this nationwide population-based study, people with type 2 diabetes and migraines were found to be at a significantly higher risk for major cardiovascular events, including MI, IS, and CVD. The risk of MI and CVD significantly increased with the presence of aura symptoms among patients with migraine.

**Supplementary Information:**

The online version contains supplementary material available at 10.1186/s12933-022-01705-3.

## Introduction

Type 2 diabetes mellitus (T2DM) is a chronic condition that develops when the body cannot effectively produce and utilize insulin [1]. T2DM is prevalent worldwide and causes a substantial socioeconomic burden to patients and society [2–4]. Patients with T2DM have a two to four times increased risk of death and cardiovascular outcomes compared to the general population [5]. While traditional vascular risk factors, including hypertension, hyperlipidemia, or obesity, are known to be associated with an increased risk for adverse cardiovascular outcomes in patients with T2DM, the impact of non- traditional risk factors, such as migraine, are yet to be determined [6]. Migraine is one of the most common primary headache disorders, affecting up to 20% of the general population [7]. One third of patients with migraine report aura symptoms, including visual disturbances, due to changes in brain activity [8]. Migraine has been associated with an increased risk of cardio-cerebrovascular disorders, including stroke and coronary artery disease [9]. Potential mechanisms for the increased risk of cardiovascular diseases in patients with migraine include endothelial dysfunction [10], inflammatory processes [11], genetic predisposition to migraine, and vascular risk factors [12] among others. Furthermore, migraine is also known to be associated with cardiovascular risk factors, including T2DM, hypertension, and obesity [13, 14]. While numerous previous epidemiological studies have already revealed that migraine with or without aura is an established risk factor for cardiovascular diseases [9, 15–18], the impact of migraine on cardiovascular outcomes in patients with T2DM has not been studied in a large cohort.

Therefore, we investigated the association between migraine and major cardiovascular outcomes, including myocardial infarction (MI), ischemic stroke (IS), cardiovascular death (CVD), and all-cause death, in patients with T2DM using the nationwide Korean National Health Insurance Service database. In addition, we also examined whether the presence of aura symptoms differentially impacts cardiovascular outcomes in patients with T2DM.

## Methods

### Database source and study population

This nationwide population-based cohort study retrieved data from the Korean National Health Insurance Service (K-NHIS) Database. A detailed summary of the K-NHIS database has been reported previously [19, 20]. In short, the K-NHIS is the only public insurance system covering almost the entire Korean population. As of 2015, 99% of the total population of Korea was covered by the K-NHIS [21]. Therefore, this database is representative of the entire population of South Korea, which consists exclusively of East Asian people as Koreans are known to be of a single race and ethnicity [22]. Furthermore, the K-NHIS offers biannual health checkups, based on age, for all subscribers and provides demographic information, results of health checkups, structured questionnaires, and history of diagnoses according to the International Statistical Classification of Diseases Related Health Problems, Tenth Revision (ICD-10). Clinical information included body mass index (BMI); basic blood tests; and social habits, such as alcohol consumption, smoking status, and physical activities. Income levels were also collected since health insurance premiums are charged differently as per income levels [23]. We also collected mortality data from Statistics Korea as previously described [24]. The applicability and strengths of the K-NHIS database compared to other databases that include information of various blood tests, demographic findings, and socioeconomic status, have been well described in other studies [25–29]. This distinguishes the current study from other database studies. Use of the K-NHIS database is permitted if study protocols are approved by both the government’s official review committee and review board of the medical institution. The institutional review board of Hallym University Dongtan Sacred Heart Hospital approved this study and waived the requirement for informed consent because of its retrospective nature and anonymized analysis (IRB No. HDT 2022–05-019). This study followed the Strengthening the Reporting of Observational Studies in Epidemiology (STROBE) reporting guidelines.

### Study population

The K-NHIS cohort used for this study consisted of people who underwent a nationwide health checkup between January 1, 2009 and December 31, 2012. Patients who had been diagnosed previously with T2DM and those who were newly diagnosed during health checkups were included in the study. We excluded patients who (1) were < 20 years of age, (2) had a past medical history of MI or stroke, or (3) had claims of migraine diagnosis after health checkups. Patients with missing data during health checkups were excluded. In total, 2,229,598 patients with T2DM were included in the main analysis. The patients were followed upon from January 1, 2009 to December 2018, and the data were analyzed between August 1, 2020 and October 31, 2020.

### Definitions of type 2 diabetes mellitus and migraine

T2DM was defined as follows: (1) having at least one claim per year for a prescription of antidiabetic medication under ICD-10-CM codes (i.e., E11–14, which excludes patients with type 1 diabetes) from the insurance claims data; or (2) not under ICD-10 E11-14 codes neither a prescription of oral hypoglycemic agents (OHA) or insulin, but having a fasting blood glucose (FBG) ≥ 126 mg/dL in the general health checkups. OHA included metformin, sulfonylureas, meglitinides, dipeptidyl peptidase 4 (DPP-4) inhibitors, thiazolidinediones, and α-glucosidase inhibitors. This definition of T2DM has been reported in previous studies based on the K-NHIS database [26, 30, 31]. Medication status was assessed at the baseline year, and duration of T2DM was measured from the first diagnosis to the index date. We defined severe diabetes as having: more than 5 years of T2DM, using insulin, and using 3 or more types of OHA.

**Fig. 1:**
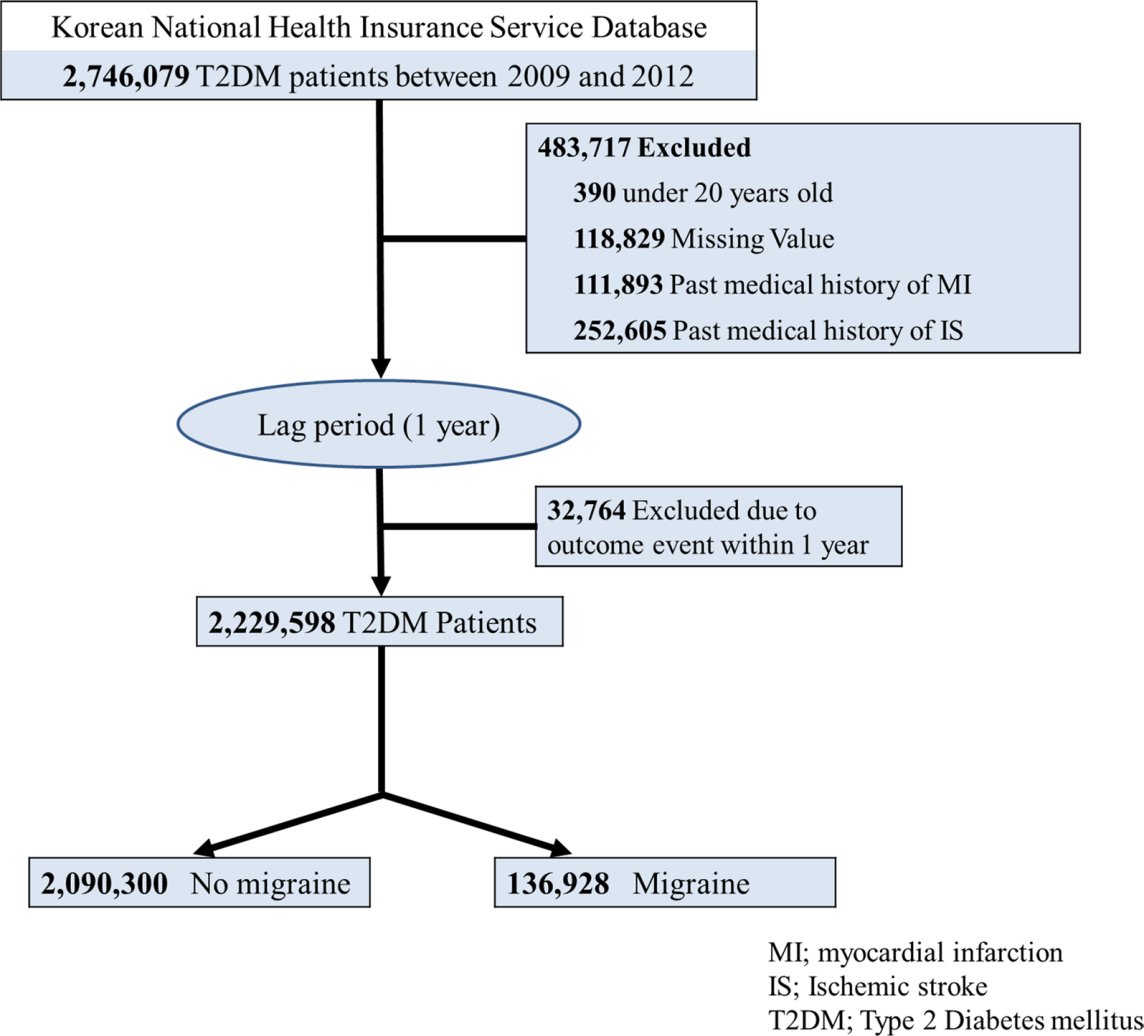
Flowchart of study population selection

We identified patients ≥ 20 years of age with migraine using the diagnostic ICD-10 code G43. Migraine with aura was defined with ICD-10 code G43.1 at least once within three years before the health check-up. Migraine without aura was defined with ICD-10 code G43.X at least once within three years before the health check-up. We set a 1-year lag-period to prevent immortal time bias for the outcomes, and a total of 32,764 patients were excluded (Fig. 1).

### Definitions of covariates and outcome variables

Demographic data, including age, sex, body weight, height, waist circumference, and previous history of vascular risk factors such as hypertension and dyslipidemia, were obtained. We also collected blood pressure and laboratory data, including liver function tests (aspartate aminotransferase [AST], alanine aminotransferase [ALT], gamma-glutamyl transferase [rGTP]), kidney function tests (glomerular filtration rate [GFR]), and lipid profiles (total cholesterol, high-density lipoprotein [HDL], low-density lipoprotein [LDL], and triglyceride). Data on lifestyle behaviors, including alcohol consumption, physical activity, and smoking habits, were collected using a self-reported questionnaire. Specifically, the average alcohol intake per day (g/day) was analyzed to evaluate alcohol consumption. Regular exercise was regarded as high-intensity physical activity (extreme shortness of breath for > 20 min per session, ≥ 3 days per week) and/or moderate-intensity physical activity (shortness of breath for > 30 min per session, ≥ 5 days per week) [32, 33]. Low-income level was defined as the composite of the lowest quartile of yearly income, as previously reported [30].

We defined newly diagnosed cardiovascular events as MI, IS, CVD, and all-cause death as study endpoints. These endpoints were defined based on ICD-10-CM claim codes with additional conditions. MI was defined as ≥ 1 claim under ICD-10 codes I21 or I22 during hospitalization or ≥ 2 claims under those codes. IS was defined as the presence of ICD-10 codes I63 or I64 during hospitalization with claims for brain imaging (magnetic resonance imaging or computed tomography) according to previous reports [34–36]. Data on the date of death were obtained from the K-NHIS database. The final follow-up was conducted in December 2018.

### Statistical analysis

Data are presented as means ± standard deviation (SD) or medians with interquartile ranges for continuous variables and numbers and frequencies for categorical variables. For continuous variables, student’s t-tests or Mann–Whitney U tests were used, as appropriate. For categorical variables, chi-square tests or Fisher’s exact tests were used, as appropriate. Multivariate Cox proportional hazard regression analyses were used to estimate hazard ratios (HR) and 95% confidence intervals (CI) for the association between migraine and cardiovascular outcomes (MI, IS, CVD, and all-cause death) in patients with T2DM. Four models were used for multivariate analysis—(1) adjusted for age and sex; (2) model 1 plus additionally adjusted for smoking status, alcohol consumption status, regular physical activity, and household income level; (3) model 2 plus additionally adjusted for history of hypertension, dyslipidemia, and BMI (calculated as weight in kilograms divided by height in meters squared); and (4) model 3 plus additionally adjusted for duration of T2DM and use of insulin and/or OHA. All analyses were 2-sided, and a *P*-value ≤ 0.05 was considered statistically significant. All statistical analyses were performed using SAS (version 9.4; SAS Institute Inc., Cary, NC, USA).

### Data and resource availability

All raw data were accessible from the designated terminals approved by the K-NHIS. Upon reasonable request, data are available through approval and oversight by the K-NHIS.

## Results

### Baseline characteristics of the study population

We identified 2,746,079 patients diagnosed with T2DM during general health checkups between January 1, 2009 and December 31, 2012. We excluded participants who were < 20 years of age (n = 390) or had a history of MI (n = 111,893) or IS (n = 252,605) before their general health checkup. After excluding patients with missing values, 2,229,598 patients with T2DM were included in the analysis. The control group (n = 2,093,300) included patients with diabetes without claims for migraine diagnosis (Fig. 1). The baseline demographic and clinical characteristics are summarized in Table 1. Migraine was diagnosed in 6.1% (n = 136,298) of the study population and was more common in women than in men (62.68% male in the non-migraine group vs. 62.64% female in the migraine group, *P* < 0.0001). From the basic demographic findings, patients with migraine were older; had higher frequency of hypertension, dyslipidemia; and were less likely to smoke or drink alcohol. The overall laboratory results and BMI did not show clinically significant differences between the two groups.

**Table 1:** Demographic and Clinical characteristics of study participants according to the presence of migraine

	Migraine		P-value
	No	Yes	
N (%)	2,093,300 (93.9)	136,298 (6.1)	
Age	55.86 ± 12.17	58.95 ± 11.9	< 0.0001
Sex,male	1,312,010 (62.68)	50,922 (37.36)	< 0.0001
Income low, 25%	483,179 (23.08)	33,469 (24.56)	< 0.0001
Hypertension	1,105,993 (52.83)	79,621 (58.42)	< 0.0001
Dyslipidemia	812,758 (38.83)	63,525 (46.61)	< 0.0001
DM duration ≥ 5yrs	581,845 (27.8)	40,309 (29.57)	< 0.0001
Use of Insulin	164,935 (7.88)	13,629 (10)	< 0.0001
OHA ≥ 3 agents	284,008 (13.57)	21,579 (15.83)	< 0.0001
Smoking			< 0.0001
Non-smoker	1,114,591 (53.25)	97,732 (71.7)	
Ex-smoker	391,666 (18.71)	16,930 (12.42)	
Current smoker	587,043 (28.04)	21,636 (15.87)	
Drinking Habits			< 0.0001
Non-drinker	1,122,378 (53.62)	96,836 (71.05)	
Mild-drinker	741,745 (35.43)	31,486 (23.1)	
Heavy-drinker	229,177 (10.95)	7,976 (5.85)	
Regular Exercise	438,841 (20.96)	25,934 (19.03)	< 0.0001
Height	163.12 ± 9.2	159.18 ± 9.05	< 0.0001
Weight	66.95 ± 11.97	64.09 ± 11.54	< 0.0001
BMI	25.07 ± 3.4	25.21 ± 3.47	< 0.0001
Waist Circumference	85.31 ± 8.65	84.84 ± 8.82	< 0.0001
Systolic BP	128.89 ± 15.74	127.95 ± 15.48	< 0.0001
Diastolic BP	79.24 ± 10.27	78.37 ± 9.93	< 0.0001
Cholesterol	198.13 ± 42.06	199.17 ± 42.8	< 0.0001
HDL	52.1 ± 21.94	52.66 ± 23.44	< 0.0001
LDL	112.29 ± 40.76	114.08 ± 41.37	< 0.0001
GFR	84.51 ± 31.98	80.4 ± 30.53	< 0.0001
^a^AST	26.37 (26.36–26.39)	25.7 (25.64–25.76)	< 0.0001
^a^ALT	26.79 (26.76–26.81)	25.41 (25.34–25.49)	< 0.0001
^a^RGTP	37.95 (37.91–37.99)	32.36 (32.23–32.49)	< 0.0001
^a^Triglyceride	146.85 (146.74–146.97)	143.44 (143.02–143.86)	< 0.0001

*DM* diabetes mellitus, *OHA* oral hypoglycemic agent, physical activity, *BMI* body mass index, *CKD* chronic kidney disease, *BP* blood pressure, *HDL* high density lipoprotein, *LDL* low density liprotein, *GFR* glomerular filtration rate, *AST*: aaspartate aminotransferase, *ALT* alanine aminotransferase, *RGTP* gamma-glutamyltransferase.

^a^Geometric means(95% Confidence Intervals)

### Association between migraine and cardiovascular outcomes

**Fig. 2:**
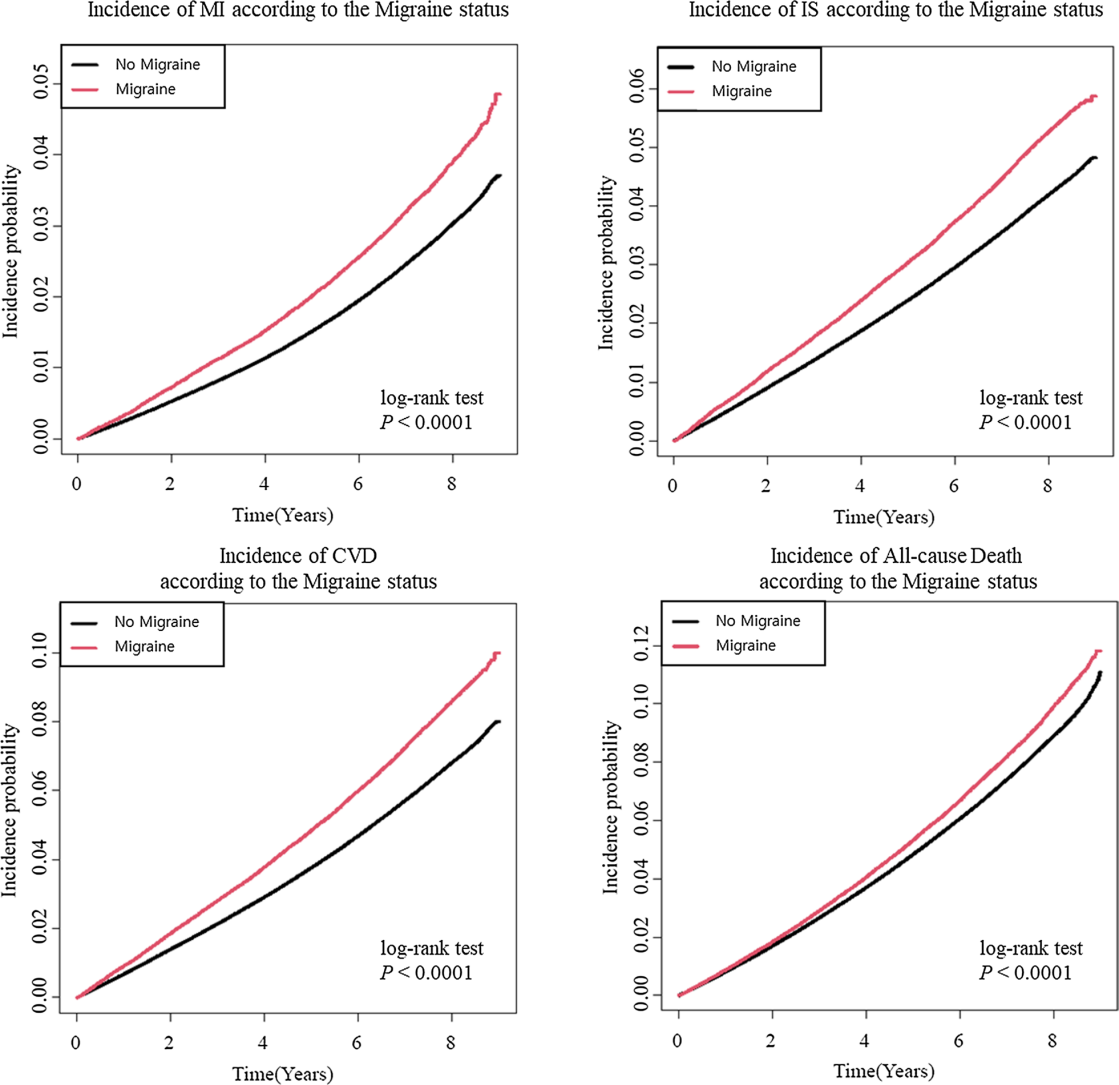
Incidence probability of MI, IS, CVD and all-cause mortality in diabetic patients according to the presence of migraine

During a median follow-up of 7.15 years of patients with T2DM, 57,116 cases (2.6%) of MI, 80,949 cases (3.6%) of IS, 130,862 cases (5.9%) of CVD, and 175,360 cases (7.9%) of all-cause death occurred. The diagnosis of migraine was significantly associated with an increased risk of MI (HR: 1.303, 95% CI 1.264–1.343), IS (HR: 1.26, 95% CI 1.228–1.294), and CVD (HR: 1.277, 95% CI 1.251–1.304) but not for all-cause death. The results remained significant after adjusting for covariates, including age, sex, BMI, alcohol intake, smoking status, physical activity, economic status, history of hypertension, dyslipidemia, duration, and severity of T2DM. (MI, adjusted HR [aHR]: 1.182, 95% CI 1.146–1.219; IS, aHR: 1.111, 95% CI 1.082–1.14; CVD, aHR: 1.143, 95% CI 1.12–1.167) (Table 2). The cumulative incidence function curves show that the differences in the incidence of MI, IS, and CVD became more prominent with increasing age (Fig. 2).

**Table 2:** Risk for Myocardial infarction (MI), Ischemic stroke (IS), cardiovascular death (CVD), and all-cause mortality according to the presence of migraine

	N	Event	RATE	HR Model^a^	aHR Model 1^b^	aHR Model 2^c^	aHR Model 3^d^	aHR Model 4^e^
Migraine		MI						
No	2,093,300	52,662	3.65	1 (Ref.)	1 (Ref.)	1 (Ref.)	1 (Ref.)	1 (Ref.)
Yes	136,298	4,454	4.76	**1.303 (1.264,1.343)**	**1.198 (1.161,1.235)**	**1.192 (1.156,1.230)**	**1.184 (1.148,1.221)**	**1.182 (1.146,1.219)**
		IS						
No	2,093,300	74,838	5.22	1 (Ref.)	1 (Ref.)	1 (Ref.)	1 (Ref.)	1(Ref.)
Yes	136,298	6,111	6.58	**1.260 (1.228,1.294)**	**1.112 (1.084,1.142)**	**1.114 (1.086,1.144)**	**1.111 (1.083,1.141)**	**1.111(1.082,1.140)**
		CVD						
No	2,093,300	120,888	8.51	1 (Ref.)	1 (Ref.)	1 (Ref.)	1 (Ref.)	1(Ref.)
Yes	136,298	9,974	10.87	**1.277 (1.251,1.304)**	**1.15 (1.126,1.173)**	**1.149 (1.125,1.173)**	**1.144 (1.120,1.168)**	**1.143(1.120,1.167)**
		Death						
No	2,093,300	163,504	11.25	1 (Ref.)	1 (Ref.)	1 (Ref.)	1 (Ref.)	1(Ref.)
Yes	136,298	11,856	12.53	**1.113 (1.092,1.134)**	1.014 (0.995,1.033)	1.015 (0.996,1.034)	**1.019 (1.001,1.039)**	1.01(0.991,1.029)

Bold fonts indicate statistically significance

*HR* hazard ratio, *aHR* adjusted hazard ratio, *MI* myocardial infarction, *IS* ischemic stroke, *CVD* Cardiovascular death

^a^HR Model: Non-adjusted;

^b^Model 1: age and sex-adjusted;

^c^Model 2: age, sex, smoking, alcohol consumption, regular physical activity, and low-income adjusted;

^d^Model 3: Model 2 + hypertension, dyslipidemia, and body mass index adjusted;

^e^Model 4: Model 3 + duration of diabetes, use of insulin, and more than 3 agents of oral antihyperglycemic agent adjusted

**Fig. 3:**
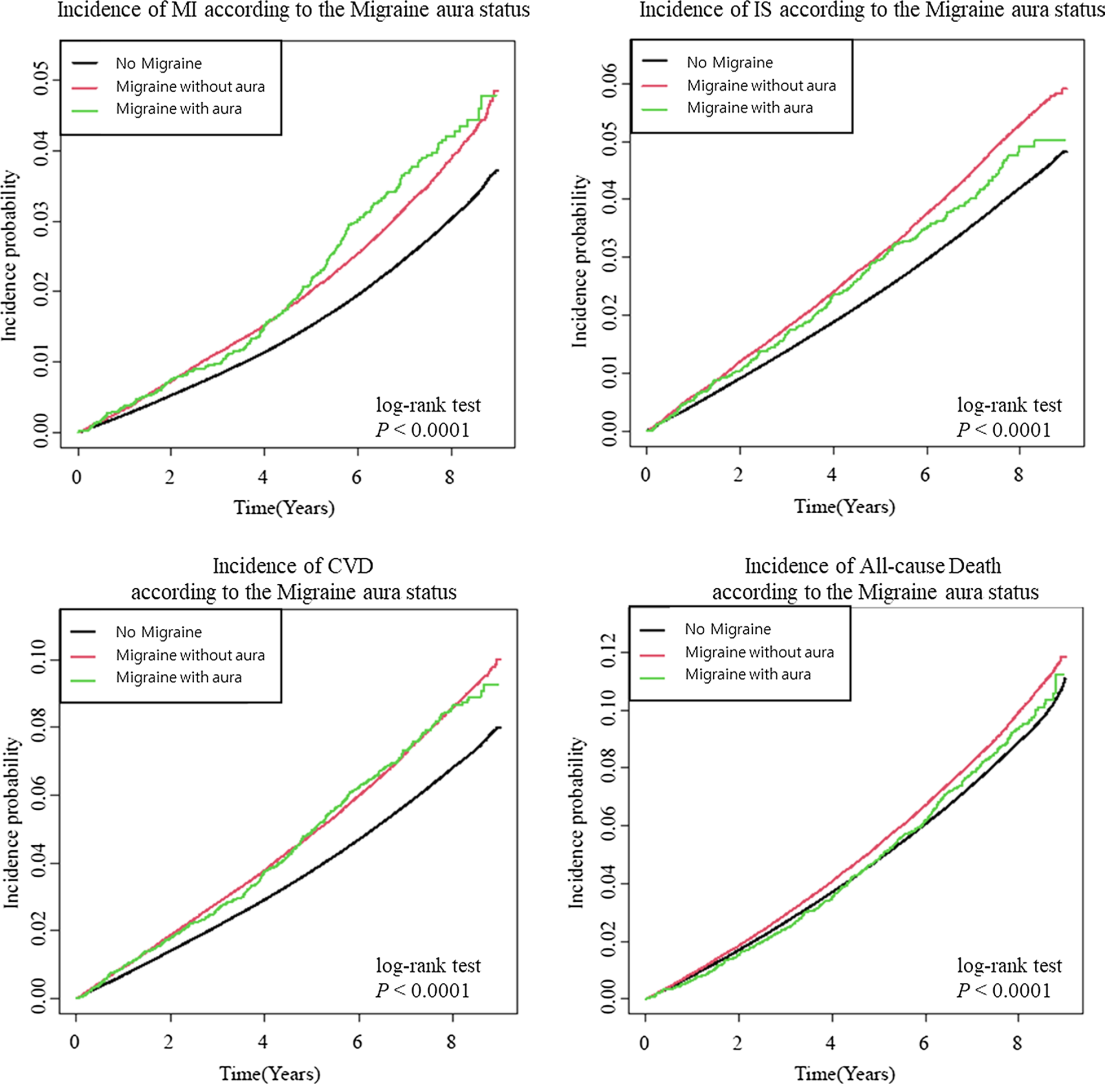
Incidence probability of MI, IS, CVD and all-cause mortality in diabetic patients according to the presence of migraine with or without aura

To determine whether the prognostic effects of migraine were modified by the presence of aura symptoms, we stratified the patients with migraine into two groups: Patients with or without aura. Regardless of the aura status, migraine was consistently associated with higher risks of MI and CVD. In general, the aHRs were higher for migraine with aura than without aura for MI (without aura, aHR: 1.173, 95% CI 1.137–1.211; with aura, aHR: 1.354, 95% CI 1.191–1.539) and CVD (without aura, aHR: 1.140, 95% CI 1.116–1.164; with aura, aHR: 1.204, 95% CI 1.101–1.317). However, while patients of migraine without aura had an increased risk of IS, those with aura did not demonstrate a significant association with the risk of IS (aHR: 1.112, 95% CI 1.083–1.143; and aHR: 1.077, 95% CI 0.956–1.214 respectively) (Table 3 and Fig. 3).

**Table 3:** Subgroup analysis according to the presence of aura and risk for Myocardial infarction (MI), Ischemic stroke (IS), cardiovascular death (CVD) and all-cause mortality

	N	Event	RATE	^a^HR Model	aHR Model 1	aHR Model 2	aHR Model 3	aHR Model 4
		MI						
No	2,093,300	52,662	3.65	1 (Ref.)	1 (Ref.)	1 (Ref.)	1 (Ref.)	1 (Ref.)
Without Aura	129,682	4,220	4.74	**1.297 (1.257,1.338)**	**1.189 (1.152,1.227)**	**1.184 (1.147,1.222)**	**1.175 (1.139,1.213)**	**1.173 (1.137,1.211)**
Aura	6,616	234	5.20	**1.433 (1.260,1.629)**	**1.380 (1.214,1.569)**	**1.371 (1.205,1.558)**	**1.36 (1.196,1.546)**	**1.354 (1.191,1.539)**
		**IS**						
No	2,093,300	74,838	5.22	1 (Ref.)	1 (Ref.)	1 (Ref.)	1 (Ref.)	1 (Ref.)
Without Aura	129,682	5,842	6.61	**1.266 (1.232,1.300)**	**1.114 (1.085,1.144)**	**1.116 (1.087,1.146)**	**1.113 (1.083,1.143)**	**1.112 (1.083,1.143)**
Aura	6,616	269	6.01	**1.153 (1.023,1.299)**	1.079 (0.957,1.216)	1.081 (0.959,1.218)	1.081 (0.959,1.218)	1.077 (0.956,1.214)
		**CVD**						
No	2,093,300	120,888	8.51	1 (Ref.)	1 (Ref.)	1 (Ref.)	1 (Ref.)	1 (Ref.)
Without Aura	129,682	9,495	10.87	**1.277 (1.251,1.304)**	**1.147 (1.123,1.171)**	**1.146 (1.122,1.170)**	**1.141 (1.117,1.165)**	**1.140 (1.116,1.164)**
Aura	6,616	479	10.84	**1.278 (1.168,1.398)**	**1.214 (1.109,1.328)**	**1.211 (1.107,1.325)**	**1.208 (1.105,1.322)**	**1.204 (1.101,1.317)**
		**Death**						
No	2,093,300	163,504	11.25	1 (Ref.)	1 (Ref.)	1 (Ref.)	1 (Ref.)	1 (Ref.)
Without Aura	129,682	11,322	12.57	**1.116 (1.095,1.138)**	1.013 (0.994,1.032)	1.014 (0.994,1.033)	1.018 (0.999,1.038)	1.009 (0.990,1.029)
Aura	6,616	534	11.72	1.045 (0.960,1.137)	1.037 (0.952,1.129)	1.037 (0.953,1.129)	1.043 (0.958,1.136)	1.029 (0.945,1.120)

Bold fonts indicate statistically significance

*HR* hazard ratio, *aHR* adjusted hazard ratio, *MI* myocardial infarction, *IS* ischemic stroke, *CVD* Cardiovascular death

^a^HR Model: Non-adjusted;

^b^Model 1: age and sex-adjusted;

^c^Model 2: age, sex, smoking, alcohol consumption, regular physical activity, and low-income adjusted;

^d^Model 3: Model 2 + hypertension, dyslipidemia, and body mass index adjusted;

^e^Model 4: Model 3 + duration of diabetes, use of insulin, and more than 3 agents of oral antihyperglycemic agent adjusted

### Subgroup analysis for cardiovascular outcomes

To determine whether the associations between migraine and the risk of cardiovascular outcomes were affected by covariates, we stratified the patients according to their age (20–65 years versus > 65 years), sex, current smoking, alcohol consumption, hypertension, dyslipidemia, obesity, use of insulin, duration of diabetes, and more than three categories of OHA. While the trends shown in the main analyses were mostly consistent in the subgroup analyses, patients with migraine who did not drink alcohol had a significantly higher risk of IS, and patients who were older or obese had a significantly higher risk for all-cause deaths (Additional file 1: Tables S1, S2).

## Discussion

In this study, we investigated the association between migraines and major cardiovascular outcomes in patients with T2DM. The main findings are as follows. First, migraine prevalence in patients with T2DM was 6.1%, which was not significantly different from the average Korean patients with migraine, previously estimated at approximately 6%. Second, migraine was associated with a higher risk of cardiovascular outcomes, including MI, IS, and CVD in these patients. Migraine with aura was significantly associated with increased development of MI and CVD.

Several studies have shown an association between migraines and major cardiovascular outcomes. A recent Danish population study showed that migraine is associated with an increased risk of MI, IS, hemorrhagic stroke, venous thromboembolism, and arrhythmias, including atrial fibrillation and atrial flutter; the associations became stronger with the presence of aura and in women compared to men [37]. Further, a 10-year follow-up study conducted in 2004 found that angina was approximately three times more frequent in migraine with auras [38]. However, this study did not reveal an association of migraine with an increase in coronary heart disease. Previous studies have shown a relationship between migraine and IS [16, 17], and a meta-analysis demonstrated an association between migraine and IS (overall pooled effect: 2.04, 95% CI 1.72–2.43). [39]

It is well known that the presence of aura is associated with even higher risk of cardiovascular outcomes in patients with migraine. A Women's Health Study conducted in 2006 showed that major coronary heart disease, including MI, occurred more frequently in a migraine group with aura compared to those with migraine without aura (multivariable-adjusted HR: 2.08, 95% CI 1.30–3.31, *P* = 0.002) [16]. Another study by the same investigators showed similar results in men (multivariable-adjusted HR: 1.42, 95% CI 1.15–1.77, *P* < 0.001) [17], and another study demonstrated that migraine diagnosis irrespective of the presence of aura was associated with MI, regardless of sex (adjusted HR: 2.2, 95% CI 1.7–2.8) [40]. A study, published in 2020, found that female patients with migraine with aura had a higher adjusted incidence rate of CVD compared with women with migraine without aura (incidence rate per 1000 person-years: 3.36; 95% CI 2.72–3.99 vs. 2.11; 95% CI 1.98–2.24 respectively, *P* < 0.001) [41]. This is consistent with the results of our study, which found that presence of aura was associated with higher aHRs compared to the migraine without aura groups for MI and CVD.

Another consideration is that the migraine with aura group did not show an increased risk for IS in our study cohorts. A previous study reported that stroke and transient ischemic attack were 2.81 and 4.28 times higher in patients with migraine with aura compared to those without aura, respectively [42]. Other studies in women found that migraine with aura increased the incidence of IS after adjusting for multiple major CVD risks [15, 16, 18]. These studies also stated that there was no association with stroke in patients without aura; however, in some studies in men, an association was found only in patients younger than 55 years [17]. Therefore, in most studies, stroke increased in patients with migraine with aura. In this study, less than 5% of the patients with migraine were diagnosed with aura, which is relatively less frequent compared to 25% in the general migraine population [43]. A study has shown that in female patients with T2DM, the prevalence of migraine tended to decrease before the diagnosis of T2DM [44]. However, no studies have yet reported the prevalence of aura symptoms in T2DM population. Further, this study cannot conclude that migraine with aura is less frequent in patients with T2DM as the diagnosis of migraine was solely based on the ICD-10 diagnosis-based claim data. Further, the non-significant association between migraine with aura and stroke occurrence may be due to insufficient sample size of migraine with aura patients and relatively less accurate diagnosis of migraine with aura. Although our study did not show any differences in overall incidence of IS in the migraine with aura group, a subgroup analysis according to sex showed a consistent difference in women with aura. In addition, a larger difference was shown depending on the presence of aura, showing similar results to other studies.

Various diseases are already known to be associated with migraine including stroke, sub-clinical vascular lesions, coronary heart disease, hypertension, patent foramen ovale, mental illness, restless leg syndrome, obesity, epilepsy, and asthma [14]. Some pathological mechanisms, such as endothelial dysfunction and vascular pathology [45] with elevated vascular biomarkers [46, 47], have been reported for migraine, but they are not well understood; therefore, further research is required in the future.

This study had several limitations. First, despite its large and well-controlled study design, it was an observational study. We were unable to exclude the possibility of unmeasured confounders. In particular, information on the use of anti-migraine medications or heart function, such as ejection fraction, was not available. Second, the diagnosis of migraine with aura may not have been accurate. As in other K-NHIS-based studies, this study was based on insurance claims with ICD-10 codes for migraine. According to the K-NHIS guidelines, brain image evaluation is insured depending on the presence or absence of aura, which makes ICD-10 dependent criteria relatively less reliable. Thus, the diagnosis of migraine may be less valid compared to the clinic or hospital-based studies using structured questionnaire and neurologists’ diagnosis. Third, the K-NHIS database consists exclusively of Far-East Asian people, and the results from this study cannot be generalized to other racial and ethnic groups. Despite these limitations, this nationwide population cohort included a large number of participants with long-term follow-up that reflected real-world practice.

## Conclusion

In this large Korean nationwide cohort study of patients with T2DM, migraine was associated with significantly increased risks of cardiovascular events such as MI, IS, and cardiovascular death. The MI and CVD risk significantly increased in the presence of migraine with aura. Further studies on the incidence of cardio-cerebrovascular disease according to subgroups, such as aura symptoms and sex in patients with migraine, are needed.

## Supplementary Information

**Additional file 1: Table S1**. Subgroup analyses according to the age, sex, smoking, alcohol habitats, hypertension, dyslipidemia, obesity, use of insulin, duration of diabetes mellitus (DM) and number of antihyperglycemic agent group and risk for Myocardial infarction (MI) and Ischemic stroke (IS). **Table S2.** Subgroup analyses according to the age, sex, smoking, alcohol habitats, hypertension, dyslipidemia, obesity, use of insulin, duration of diabetes mellitus (DM) and number of antihyperglycemic agent group and risk for Cardiovascular Death (CVD) and all cause of death.

## Data Availability

Anonymized dataset for this study is publicly available from the Korean National Health Insurance Sharing Service and can be accessed at https://nhiss.nhis.or.kr/bd/ab/bdaba000eng.do.
