# Supplementary material for: Associations between migraine and major cardiovascular events in type 2 diabetes mellitus
Source: Cardiovasc Diabetol. 2022 Dec 9;21:275. doi: 10.1186/s12933-022-01705-3 (PMC9737987; doi:10.1186/s12933-022-01705-3)
Supplement: Supplementary file 1 — Additional file 1: Table S1. Subgroup analyses according to the age, sex, smoking, alcohol habitats, hypertension, dyslipidemia, obesity, use of insulin, duration of diabetes mellitus (DM) and number of antihyperglycemic agent group and risk for Myocardial infarction (MI) and Ischemic stroke (IS). Table S2. Subgroup analyses according to the age, sex, smoking, alcohol habitats, hypertension, dyslipidemia, obesity, use of insulin, duration of diabetes mellitus (DM) and number of antihyperglycemic agent group and risk for Cardiovascular Death (CVD) and all cause of death. [file 12933_2022_1705_MOESM1_ESM.docx]

**Supplementary Table 1. Subgroup analyses according to the age, sex, smoking, alcohol habitats, hypertension, dyslipidemia, obesity, use of insulin, duration of diabetes mellitus (DM) and number of antihyperglycemic agent group and risk for Myocardial infarction (MI) and Ischemic stroke (IS).**

| **Subgroup** | **Migraine** | **N** | **MI** | **Rate** | **^a^aHR Model** | **p for interaction** | **IS** | **Rate** | **aHR Model** | **p for**  **interaction** |
| --- | --- | --- | --- | --- | --- | --- | --- | --- | --- | --- |
| **Age** |  |  |  |  |  |  |  |  |  |  |
| <65 | No | 1,567,297 | 28,575 | 2.61 | 1(Ref.) | 0.634 | 34,592 | 3.17 | 1(Ref.) | 0.057 |
|  | Yes | 89,359 | 1929 | 3.09 | 1.171(1.118,1.227) |  | 2,128 | 3.42 | 1.073(1.027,1.121) |  |
| ≥65 | No | 526,003 | 24,087 | 6.92 | 1(Ref.) |  | 40,246 | 11.74 | 1(Ref.) |  |
|  | Yes | 46,939 | 2525 | 8.10 | 1.189(1.141,1.239) |  | 3,983 | 12.99 | 1.131(1.095,1.169) |  |
| **Sex** |  |  |  |  |  |  |  |  |  |  |
| Male | No | 1,312,010 | 32,706 | 3.64 | 1(Ref.) | 0.148 | 45,392 | 5.08 | 1(Ref.) | 0.036 |
|  | Yes | 50,922 | 1676 | 4.87 | 1.149(1.094,1.207) |  | 2,228 | 6.52 | 1.072(1.028,1.119) |  |
| Female | No | 781,290 | 19,956 | 3.67 | 1(Ref.) |  | 29,446 | 5.46 | 1(Ref.) |  |
|  | Yes | 85,376 | 2778 | 4.70 | 1.204(1.157,1.252) |  | 3,883 | 6.62 | 1.136(1.099,1.175) |  |
| **Smoking** |  |  |  |  |  |  |  |  |  |  |
| No | No | 1,506,257 | 36,860 | 3.53 | 1(Ref.) | 0.665 | 54,323 | 5.24 | 1(Ref.) | 0.032 |
|  | Yes | 114,662 | 3651 | 4.62 | 1.185(1.146,1.227) |  | 5,165 | 6.58 | 1.125(1.093,1.158) |  |
| Yes | No | 587,043 | 15,802 | 3.96 | 1(Ref.) |  | 20,515 | 5.17 | 1(Ref.) |  |
|  | Yes | 21,636 | 803 | 5.55 | 1.165(1.085,1.251) |  | 946 | 6.58 | 1.041(0.975,1.111) |  |
| **Drink** |  |  |  |  |  |  |  |  |  |  |
| No | No | 1,122,378 | 33,578 | 4.35 | 1(Ref.) | 0.141 | 46,265 | 6.03 | 1(Ref.) | <0.001 |
|  | Yes | 96,836 | 3465 | 5.22 | 1.167(1.127,1.209) |  | 4,845 | 7.36 | 1.144(1.111,1.179) |  |
| Yes | No | 970,922 | 19,084 | 2.85 | 1(Ref.) |  | 28,573 | 4.29 | 1(Ref.) |  |
|  | Yes | 39,462 | 989 | 3.65 | 1.233(1.156,1.314) |  | 1,266 | 4.69 | 1.005(0.950,1.063) |  |
| **HTN** |  |  |  |  |  |  |  |  |  |  |
| No | No | 987,307 | 18,255 | 2.67 | 1(Ref.) | 0.013 | 23,310 | 3.42 | 1(Ref.) | 0.995 |
|  | Yes | 56,677 | 1426 | 3.64 | 1.25(1.184,1.320) |  | 1,671 | 4.28 | 1.111(1.057,1.167) |  |
| Yes | No | 1,105,993 | 34,407 | 4.54 | 1(Ref.) |  | 51,528 | 6.86 | 1(Ref.) |  |
|  | Yes | 79,621 | 3,028 | 5.58 | 1.151(1.109,1.195) |  | 4,440 | 8.25 | 1.111(1.077,1.146) |  |
| **DYS** |  |  |  |  |  |  |  |  |  |  |
| No | No | 1,280,542 | 29,744 | 3.38 | 1(Ref.) | 0.325 | 44,838 | 5.13 | 1(Ref.) | 0.460 |
|  | Yes | 72,773 | 2,179 | 4.38 | 1.163(1.114,1.215) |  | 3,305 | 6.69 | 1.121(1.082,1.161) |  |
| Yes | No | 812,758 | 22,918 | 4.08 | 1(Ref.) |  | 30,000 | 5.36 | 1(Ref.) |  |
|  | Yes | 63,525 | 2,275 | 5.21 | 1.200(1.149,1.253) |  | 2,806 | 6.46 | 1.099(1.057,1.143) |  |
| **BMI** |  |  |  |  |  |  |  |  |  |  |
| <25 | No | 1,073,637 | 28,309 | 3.86 | 1(Ref.) | 0.213 | 41,513 | 5.70 | 1(Ref.) | 0.017 |
|  | Yes | 68,063 | 2,290 | 4.95 | 1.160(1.111,1.210) |  | 3,223 | 7.02 | 1.079(1.041,1.118) |  |
| ≥25 | No | 1,019,663 | 24,353 | 3.44 | 1(Ref.) |  | 33,325 | 4.73 | 1(Ref.) |  |
|  | Yes | 68,235 | 2164 | 4.58 | 1.206(1.154,1.260) |  | 2,888 | 6.15 | 1.149(1.106,1.194) |  |
| **Insulin use** |  |  |  |  |  |  |  |  |  |  |
| No | No | 1,928,365 | 44,897 | 3.37 | 1(Ref.) | 0.264 | 64,196 | 4.84 | 1(Ref.) | 0.036 |
|  | Yes | 122,669 | 3,637 | 4.30 | 1.172(1.133,1.213) |  | 5,016 | 5.97 | 1.097(1.066,1.130) |  |
| Yes | No | 164,935 | 7,765 | 7.16 | 1(Ref.) |  | 10,642 | 9.91 | 1(Ref.) |  |
|  | Yes | 13,629 | 817 | 9.13 | 1.226(1.141,1.318) |  | 1,095 | 12.32 | 1.181(1.109,1.256) |  |
| **DM duration** |  |  |  |  |  |  |  |  |  |  |
| <5yrs | No | 1,511,455 | 30,670 | 2.95 | 1(Ref.) | 0.057 | 42,735 | 4.13 | 1(Ref.) | 0.408 |
|  | Yes | 95,989 | 2,624 | 4.00 | 1.212(1.164,1.261) |  | 3,444 | 5.27 | 1.100(1.062,1.139) |  |
| ≥5yrs | No | 581,845 | 21,992 | 5.47 | 1(Ref.) |  | 32,103 | 8.06 | 1(Ref.) |  |
|  | Yes | 40,309 | 1,830 | 6.53 | 1.141(1.088,1.197) |  | 2,667 | 9.70 | 1.125(1.081,1.170) |  |
| **OHA ≥ 3** |  |  |  |  |  |  |  |  |  |  |
| No | No | 1,809,292 | 41,983 | 3.38 | 1(Ref.) | 0.735 | 59,371 | 4.80 | 1(Ref.) | 0.019 |
|  | Yes | 114,719 | 3,463 | 4.41 | 1.185(1.144,1.227) |  | 4,656 | 5.97 | 1.092(1.06,1.126) |  |
| Yes | No | 284,008 | 10,679 | 5.40 | 1(Ref.) |  | 15,467 | 7.89 | 1(Ref.) |  |
|  | Yes | 21,579 | 991 | 6.60 | 1.17(1.096,1.249) |  | 1,455 | 9.82 | 1.176(1.114,1.241) |  |
| Abbreviations. MI, myocardial infarction; IS, ischemic stroke; HTN, hypertension; DYS, dyslipidemia; BMI, body mass index; OHA, oral antihyperglycemic agent; aHR, adjusted hazard ratio. ^a^aHR Model: age and sex, smoking, alcohol consumption, regular physical activity, low-income, hypertension, dyslipidemia, body mass index, duration of diabetes, use of insulin, and more than 3 agents of oral antihyperglycemic agent adjusted | | | | | | | | | | |

**Supplementary Table 2. Subgroup analyses according to the age, sex, smoking, alcohol habitats, hypertension, dyslipidemia, obesity, use of insulin, duration of diabetes mellitus (DM) and number of antihyperglycemic agent group and risk for Cardiovascular Death (CVD) and all cause of death.**

| **Subgroup** | **Migraine** | **N** | **CVD** | **Rate** | ^a^**aHR Model** | **p for interaction** | **All cause death** | **Rate** | **aHR Model** | **p for**  **interaction** |
| --- | --- | --- | --- | --- | --- | --- | --- | --- | --- | --- |
| **Age** |  |  |  |  |  |  |  |  |  |  |
| <65 | No | 1,567,297 | 60,534 | 5.59 | 1(Ref.) | 0.276 | 60,381 | 5.49 | 1(Ref.) | <0.001 |
|  | Yes | 89,359 | 3,904 | 6.33 | 1.126(1.090,1.164) |  | 3,035 | 4.83 | 0.956(0.922,0.992) |  |
| ≥65 | No | 526,003 | 60,354 | 17.87 | 1(Ref.) |  | 103,123 | 29.15 | 1(Ref.) |  |
|  | Yes | 46,939 | 6,070 | 20.17 | 1.153(1.123,1.184) |  | 8,821 | 27.74 | 1.030(1.008,1.053) |  |
| **Sex** |  |  |  |  |  |  |  |  |  |  |
| Male | No | 1,312,010 | 74,281 | 8.39 | 1(Ref.) | 0.015 | 110,708 | 12.22 | 1(Ref.) | 0.221 |
|  | Yes | 50,922 | 3,701 | 10.96 | 1.107(1.071,1.145) |  | 5,446 | 15.65 | 0.998(0.971,1.025) |  |
| Female | No | 781,290 | 46,607 | 8.71 | 1(Ref.) |  | 52,796 | 9.63 | 1(Ref.) |  |
|  | Yes | 85,376 | 6,273 | 10.82 | 1.167(1.136,1.198) |  | 6,410 | 10.71 | 1.022(0.995,1.048) |  |
| **Smoking** |  |  |  |  |  |  |  |  |  |  |
| No | No | 1,506,257 | 86,413 | 8.41 | 1(Ref.) | 0.183 | 116,395 | 11.07 | 1(Ref.) | 0.172 |
|  | Yes | 114,662 | 8,305 | 10.71 | 1.150(1.125,1.177) |  | 9,699 | 12.13 | 1.017(0.996,1.038) |  |
| Yes | No | 587,043 | 34,475 | 8.78 | 1(Ref.) |  | 47,109 | 11.70 | 1(Ref.) |  |
|  | Yes | 21,636 | 1,669 | 11.77 | 1.109(1.056,1.165) |  | 2,157 | 14.72 | 0.983(0.942,1.027) |  |
| **Drink** |  |  |  |  |  |  |  |  |  |  |
| No | No | 1,122,378 | 75,403 | 9.94 | 1(Ref.) | 0.028 | 101,827 | 13.06 | 1(Ref.) | 0.057 |
|  | Yes | 96,836 | 7,831 | 12.05 | 1.158(1.131,1.185) |  | 9,168 | 13.63 | 1.021(0.999,1.043) |  |
| Yes | No | 970,922 | 45,485 | 6.88 | 1(Ref.) |  | 61,677 | 9.15 | 1(Ref.) |  |
|  | Yes | 39,462 | 2,143 | 8.01 | 1.096(1.049,1.144) |  | 2,688 | 9.83 | 0.978(0.941,1.016) |  |
| **HTN** |  |  |  |  |  |  |  |  |  |  |
| No | No | 987,307 | 39,736 | 5.87 | 1(Ref.) | 0.076 | 54,739 | 7.95 | 1(Ref.) | 0.057 |
|  | Yes | 56,677 | 2,956 | 7.64 | 1.176(1.133,1.221) |  | 3,417 | 8.64 | 0.982(0.949,1.017) |  |
| Yes | No | 1,105,993 | 81,152 | 10.93 | 1(Ref.) |  | 108,765 | 14.21 | 1(Ref.) |  |
|  | Yes | 79,621 | 7,018 | 13.23 | 1.130(1.102,1.158) |  | 8,439 | 15.32 | 1.022(1.000,1.045) |  |
| **DYS** |  |  |  |  |  |  |  |  |  |  |
| No | No | 1,280,542 | 70,828 | 8.18 | 1(Ref.) | 0.906 | 108,309 | 12.22 | 1(Ref.) | 0.012 |
|  | Yes | 72,773 | 5,184 | 10.61 | 1.142(1.11,1.175) |  | 7,044 | 13.99 | 0.991(0.967,1.015) |  |
| Yes | No | 812,758 | 50,060 | 9.04 | 1(Ref.) |  | 55,195 | 9.72 | 1(Ref.) |  |
|  | Yes | 63,525 | 4,790 | 11.1 | 1.145(1.111,1.179) |  | 4,812 | 10.87 | 1.040(1.010,1.071) |  |
| **BMI** |  |  |  |  |  |  |  |  |  |  |
| <25 | No | 1,073,637 | 66,009 | 9.14 | 1(Ref.) | 0.009 | 104,855 | 14.17 | 1(Ref.) | 0.002 |
|  | Yes | 68,063 | 5,188 | 11.44 | 1.114(1.083,1.146) |  | 7,339 | 15.68 | 0.988(0.965,1.012) |  |
| ≥25 | No | 1,019,663 | 54,879 | 7.87 | 1(Ref.) |  | 58,649 | 8.22 | 1(Ref.) |  |
|  | Yes | 68,235 | 4,786 | 10.34 | 1.176(1.142,1.212) |  | 4,517 | 9.45 | 1.050(1.018,1.082) |  |
| **Insulin use** |  |  |  |  |  |  |  |  |  |  |
| No | No | 1,928,365 | 103,727 | 7.89 | 1(Ref.) | 0.012 | 134,404 | 10.00 | 1(Ref.) | 0.050 |
|  | Yes | 122,669 | 8,195 | 9.86 | 1.130(1.105,1.156) |  | 9,493 | 11.11 | 1.020(0.998,1.041) |  |
| Yes | No | 164,935 | 17,161 | 16.23 | 1(Ref.) |  | 29,100 | 26.38 | 1(Ref.) |  |
|  | Yes | 13,629 | 1,779 | 20.54 | 1.210(1.153,1.271) |  | 2,363 | 25.78 | 0.973(0.933,1.015) |  |
| **DM duration** |  |  |  |  |  |  |  |  |  |  |
| <5yrs | No | 1,511,455 | 70,086 | 6.83 | 1(Ref.) | 0.947 | 95,820 | 9.16 | 1(Ref.) | 0.149 |
|  | Yes | 95,989 | 5,745 | 8.88 | 1.144(1.113,1.175) |  | 6,871 | 10.36 | 0.998(0.974,1.023) |  |
| ≥5yrs | No | 581,845 | 50,802 | 12.93 | 1(Ref.) |  | 67,684 | 16.62 | 1(Ref.) |  |
|  | Yes | 40,309 | 4,229 | 15.63 | 1.142(1.107,1.179) |  | 4,985 | 17.62 | 1.027(0.997,1.057) |  |
| **OHA ≥ 3** |  |  |  |  |  |  |  |  |  |  |
| No | No | 1,809,292 | 96,335 | 7.86 | 1(Ref.) | 0.096 | 131,576 | 10.50 | 1(Ref.) | 0.253 |
|  | Yes | 114,719 | 7,684 | 9.96 | 1.132(1.106,1.159) |  | 9,281 | 11.70 | 1.004(0.983,1.026) |  |
| Yes | No | 284,008 | 24,553 | 12.69 | 1(Ref.) |  | 31,928 | 15.92 | 1(Ref.) |  |
|  | Yes | 21,579 | 2,290 | 15.70 | 1.180(1.131,1.232) |  | 2,575 | 16.87 | 1.031(0.991,1.074) |  |
| Abbreviations. CVD, cardiovascular death; HTN, hypertension; DYS, dyslipidemia; BMI, body mass index; OHA, oral antihyperglycemic agent; aHR, adjusted hazard ratio. ^a^aHR Model: age and sex, smoking, alcohol consumption, regular physical activity, low-income, hypertension, dyslipidemia, body mass index, duration of diabetes, use of insulin, and more than 3 agents of oral antihyperglycemic agent adjusted | | | | | | | | | | |
